# The Apex-Beat in Children

**Published:** 1889-02

**Authors:** 


### THE APEX-BEAT IN CHILDREN.

W. von Starck has recently made a series of elaborate investigations in regard to the situation of the apex-beat in children, his conclusions being published in *Centralblatt für Klinische Medicin*, No. 34, 1888. They are as follows: 1. During the first year of life the situation of the apex-beat is frequently indeterminable. 2. Up to the fourth year it lies without the nipple line in most children; less and less frequently during the following years; and practically never after the thirteenth year. 3. It is seldom found in the mammary line in the first year; more and more frequently so until the seventh year; less often after that time; but again there at fourteen years. 4. It is never within the mammary line before the second year, and seldom before the seventh; from nine up in most cases; and almost always from thirteen up. 5. It lies in the 4th interspace almost always in the first year; less and less often there afterwards. 6. It is in the 4th and 5th interspaces seldom during the first two years; often from the third to the sixth; less often afterwards. 7. It is very seldom in the 5th interspace in the first two years; more often in the next years; from seven up in most subjects, and almost always there after thirteen years. 8. It is very seldom found in the 6th interspace in children.

